# Chronic Alcohol Abuse Alters Hepatic Trace Element Concentrations-Metallomic Study of Hepatic Elemental Composition by Means of ICP-OES

**DOI:** 10.3390/nu14030546

**Published:** 2022-01-27

**Authors:** Jacek Baj, Grzegorz Teresiński, Alicja Forma, Michał Flieger, Jędrzej Proch, Przemysław Niedzielski, Cezary Grochowski, Eliza Blicharska, Grzegorz Buszewicz, Jacek Bogucki, Dariusz Majerek, Kaja Karakuła, Marcin Czeczelewski, Jolanta Flieger

**Affiliations:** 1 Department of Anatomy, Medical University of Lublin, Jaczewskiego 4 (Collegium Anatomicum), 20-090 Lublin, Poland; michalflieeeger@gmail.com; 2 Department of Forensic Medicine, Medical University of Lublin, Jaczewskiego 8b (Collegium Pathologicum), 20-090 Lublin, Poland; grzegorz.teresinski@umlub.pl (G.T.); aforma@onet.pl (A.F.); g.buszewicz@umlub.pl (G.B.); marcin.czeczelewski@gmail.com (M.C.); 3 Department of Analytical Chemistry, Faculty of Chemistry, Adam Mickiewicz University, 89B Umultowska Street, 61-614 Poznan, Poland; jed.proch@gmail.com (J.P.); pnied@amu.edu.pl (P.N.); 4 Laboratory of Virtual Man, Medical University of Lublin, Jaczewskiego 4 (Collegium Anatomicum), 20-439 Lublin, Poland; cezary.grochowski@umlub.pl; 5 Department of Analytical Chemistry, Medical University of Lublin, Chodźki 4A, 20-093 Lublin, Poland; bayrena@o2.pl (E.B.); j.flieger@umlub.pl (J.F.); 6 Department of Organic Chemistry, Medical University of Lublin, Chodźki 4A, 20-093 Lublin, Poland; jacekbogucki@umlub.pl; 7 Department of Applied Mathematics, University of Technology, Nadbystrzycka 38D, 20-618 Lublin, Poland; d.majerek@pollub.pl; 8 Department of Psychiatry, Psychotherapy and Early Intervention, Medical University of Lublin, Głuska 1 (SPSK Nr 1), 20-439 Lublin, Poland; kaja.karakula@gmail.com

**Keywords:** alcohol use disorder, trace elements, liver, hepatic, microelements, macroelements

## Abstract

Trace element accumulation varies in different human tissues. Distribution of several elements was found to be disrupted in the case of excessive alcohol consumption, causing negative effects and exacerbation of pathological processes in the liver. In this study, we analyzed the levels and interactions between seven trace elements including calcium (Ca), cobalt (Co), chromium (Cr), copper (Cu), iron (Fe), potassium (K), and magnesium (Mg), manganese (Mn), sodium (Na), zinc (Zn), and selenium (Se) in individuals with alcohol-use disorder (AUD) and patients without AUD (control group). The liver samples were collected during autopsy from 39 individuals with AUD and 45 control subjects. Elemental composition inductively coupled plasma optical emission spectrometry (ICP-OES) after wet mineralization by nitric acid was applied for the evaluation of the samples. Positive correlations dominated in the AUD group, mainly in relation to Mg, which strongly positively correlated with Ca, Mn, Fe; K correlated with Mn and Zn, and Cu positively correlated with K and Zn. The strongest positive correlation in the AUD group was observed for the Mg-Mn pair (r = 0.87). Significant statistical differences (*p* < 0.05) between the groups concerned the average concentration of Co, Cu, Mn, and Mg, which were lower in the AUD group, and Fe, the level of which was significantly higher in the AUD group compared to the control group. Evaluation of the chronic alcohol consumption effect on the accumulation of trace elements in the liver allows a better understanding of the pathological processes taking place in this organ.

## 1. Introduction

Consuming alcoholic beverages is firmly embedded in human history and culture. Addiction resulting from excessive and chronic alcohol consumption is now a global problem. Due to its harmful effects, ethanol causes a total of 5.3% of deaths in the world and accounts for 5.1% of all diseases and injuries [1]. Occasional heavy alcohol use is defined as a pattern of consuming 60 g or more of pure ethanol at least once a month. Some countries have a high proportion (≥60%) of occasional drinkers, including Poland [2]. Between 2000 and 2019, the WHO witnessed an increase in total alcohol consumption per capita per year by 9.4 and 11.9 L of pure ethanol, respectively. Statistical analyses predict an increase to 12.7 L in 2025 [3].

Excessive and chronic alcohol consumption affects numerous metabolic pathways, causing damage to various organs, including the brain, peripheral and autonomic nerves, liver, pancreas, and heart [4]. Consequently, binge drinking leads to the progression of alcohol-use disorder (AUD), increasing the likelihood of developing alcoholic neuropathy, heart failure, and chronic pancreatitis [5,6,7,8,9,10]. The daily consumption of pure alcohol, which may cause the aforementioned clinical conditions, is estimated at 20 g and 40 g in women and men, respectively [11,12]. Polish men drink over 3 times more alcohol than women (18.7 vs. 5.6 L of pure alcohol annually), which corresponds to an average of 43.7 and 12.3 g of alcohol per day [3].

Chronic alcohol consumption disturbs the electrolyte balance and the levels of trace elements in the serum and tissues, mainly as a result of gastrointestinal dysfunction and malabsorption, which is reflected in general malnutrition [13,14]. A number of reports describe disturbances in the concentration of trace elements in various tissues as a result of chronic alcohol consumption [15,16]. The most frequently reported abnormalities are iron overload, hypomagnesemia, and selenium and zinc deficiency. Itokawa (2000) observed a decrease in the concentration of copper in the livers of rats given alcohol compared to control rats [17].

The kidneys and lungs only eliminate 2% to 10% of the absorbed alcohol, the rest is mainly metabolized by the liver. The hepatic metabolic rate of ethanol is devoid of effective feedback control. This leads to up to 90% of the displacement of normal metabolic substances by alcohol and causes metabolic imbalances [18]. Hepatocytes are primarily affected by altered levels of trace elements and electrolytes. However, there is still a small amount of research into the accumulation of trace elements in the liver, particularly among individuals with AUD.

Poles seem very vulnerable to the harmful effects of ethanol due to excessive consumption. It seems necessary to study this population in the context of micronutrients alteration and toxic elements bioaccumulation. In the study, the authors analyzed the liver content of several trace elements, including calcium (Ca), cobalt (Co), chromium (Cr), copper (Cu), iron (Fe), potassium (K), and magnesium (Mg), manganese (Mn), sodium (Na), zinc (Zn), and selenium (Se) in individuals with AUD and the controls.

The aim of the study was to identify significant differences between the study group (n = 39) and the control group (n = 45). Liver tissue samples were collected during the autopsy. The research hypothesis was as follows: chronic alcohol consumption has a statistically significant influence on the levels of elements detected in tissues collected post mortem from the liver. The evaluation of the tendency of the alteration, accumulation of elements in the liver in individuals with chronic alcoholism as compared to the controls, as well as the inter-elemental relationships between the concentrations of the examined elements in the liver, can help to identify the risks and find mechanisms of possible protective action.

## 2. Material and Methods

### 2.1. Population and Sample Characterization

Liver tissue samples were collected from the deceased during the autopsies undertaken in the Department of Forensic Medicine, Medical University of Lublin, Poland. The prosecutor’s office competent for the deceased’s place of residence consented to the collection of tissues, and the study was approved by the Bioethics Committee of the Medical University of Lublin (approval no KE-0254/181/2021). The group inclusion criteria were based on shared post-section documentation. Criteria for inclusion in the group of cases: chronic alcohol consumption reported in medical history, the result of blood alcohol concentration (BAC), collected during the autopsy, above 70 mg/dL, macroscopically changed liver tissue such as liver fibrosis, alcoholic fatty liver disease (AFLD) or alcoholic liver cirrhosis. Inclusion criteria for the control group: non-alcohol-abusers, BAC below 70 mg/dL, no documented history of alcohol disorders, liver showing no signs of liver disease at autopsy. Blood alcohol levels at autopsy were tested up to a maximum 48 h after death. BAC cut-off value on the level of 70 mg/dL was chosen in connection to the post-mortem endogenous alcohol production via the process of fermentation due to a range of micro-organisms mainly by using glucose as a substrate [19,20]. The total number of liver tissue samples was 84, among which 39 were obtained from individuals with AUD, and 45 remained a control group. The demographic characteristic of the patients’ groups is collected in Table 1.

### 2.2. Sample Collection Procedure

Liver tissue samples collection was performed by the qualified pathologists according to the proper scheme and protocol of the autopsy. Livers were removed from the abdomen cavities just after removal of the peritoneum and afterward washed with ultrapure water (Milli-Q, Millipore, Raleigh, NC, USA; resistivity: 18.2 MΩ cm). The same ultrapure water was used to wash all of the tools used during the autopsy (knives, tweezers, and scissors) in order to minimize the probability of sample contamination. The weight of a sample was 0.5–1.5 g.

### 2.3. Sample Preparation Procedure

The liver samples were collected from the sixth organ’ segment. The samples were thoroughly rinsed with deionized water, drained, and weighed. Each tissue sample was then put into sterile polypropylene containers, and, 2 mL of 65% supra pure HNO_3_ was added. Then, the sample was transferred to close Teflon containers and digested at 180 °C utilizing the microwave digestion system Mars 6 (CEM, Matthews, NC, USA). After mineralization, samples were diluted to a total volume of 10.0 mL with water.

### 2.4. Inductively Coupled Plasma Optical Emission Spectrometer (ICP-OES) Measurements

The determination of trace elements was performed by the use of an inductively coupled plasma optical emission spectrometer Agilent 5110 ICP-OES (Agilent, Santa Clara, CA, USA). The synchronous vertical dual view (SVDV) of the plasma was accomplished by using dichronic spectral combiner (DSC) technology. Thanks to that we could create axial and radial view analysis simultaneously. The analysis was repeated three times for each sample. For calibration, we used ICP commercial analytical standards (Romil, Cambridge, UK). The conditions of measurements and validation parameters were described previously [12]. The uncertainty for the whole analytical process was at the level of 20%. By comparing with reference materials, we could assess traceability. An approximate 80–120% recovery was considered as acceptable for all the determined elements. Table 2 presents the wavelengths applied for the elements during the measurements. 

### 2.5. Statistical Analysis

The collected material was analyzed using the quantitative methods including the descriptive statistics of the concentrations of specific elements in both of the studied groups. Furthermore, an analysis of the concentration distributions of macro- and microelements was conducted using kernel density estimation. Depending on the shape of the distributions of response variables, statistical tests were carried out to verify the hypothesis on the difference in the central tendencies of the concentrations of elements between groups. In the case of asymmetry of distributions, the non-parametric Mann–Whitney U test was applied. The Spearman coefficient was used to study the correlation.

## 3. Results

### 3.1. The Average Trace Elements in the Liver Samples for the Case Group and Controls

**Figure 1:**
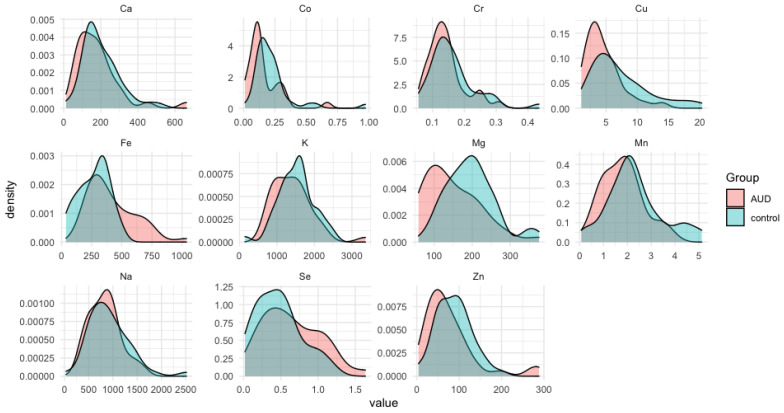
The distribution densities of the elements’ concentration in the alcohol-use disorder (AUD) and the control groups.

Based on the results of ICP-OES analyses, descriptive statistics parameters were calculated, such as average concentrations of individual metals expressed in µg/g wet weight, their range, median, standard deviation (SD), and standard error (SE). Descriptive statistics for the group of cases and controls are collected in Table 3 (Table 3). Based on the presented summary, it can be seen that the highest levels concern micronutrients from the group of macroelements such as sodium (Na), potassium (K), magnesium (Mg), calcium (Ca), and iron (Fe) in the liver tissue in both the AUD and control groups. For these elements, the highest SD values can be noticed, which indicates high individual variability in the level of macronutrients in the liver tissue. Next in terms of content are zinc (Zn) and copper (Cu). The next series deals with manganese (Mn) and selenium (Se). The lowest levels were noted for cobalt (Co) and chromium (Cr). From the median values, the following series can be constructed (in descending order) for the AUD group: K, Na, Fe, Ca, Mg, Zn, Fe, Cu, Mn, Se, Cr, Co; and for controls: K, Na, Fe, Ca, Mg, Zn, Fe, Cu, Mn, Se, Co, Cr. The distribution density of the experimental data (value) obtained for individual elements in both studied groups is presented in the graphs in Figure 1. As can be noticed the distribution was characterized by strong asymmetry in most cases.

### 3.2. Correlations of Average Trace Elements in the Liver Samples for the Case Group and Controls

**Figure 2:**
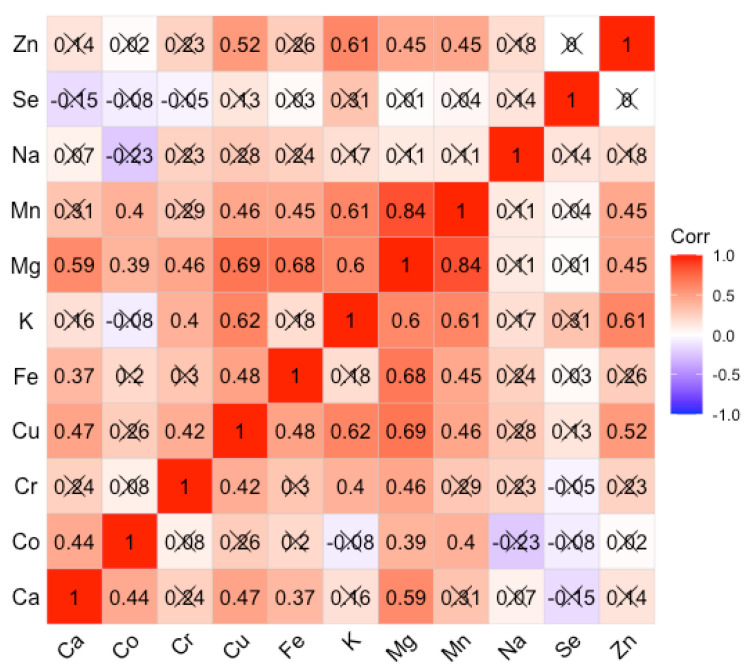
Spearman rank-order correlation matrix for AUD group. Insignificant correlation coefficients are crossed out.

**Figure 3:**
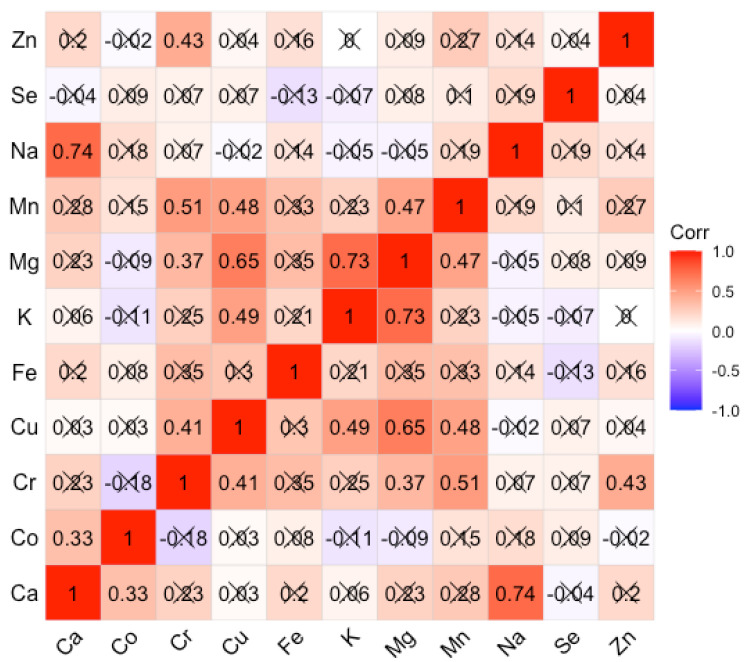
Spearman rank-order correlation matrix for control group. Insignificant correlation coefficients are crossed out.

The inter-element correlations for the AUD and control groups are expressed as the Spearman rank-order correlation coefficient. The choice of Spearman’s rank correlation coefficient was dictated by the fact that most of the elements were characterized by strong asymmetry, and the relationships between concentrations were not always linear. The coefficient values are summarized in Figures for the AUD and control groups respectively (Figure 2 and Figure 3). For the AUD group, we obtained mainly weak, positive correlations with the Spearman correlation coefficient (Spearman’s r) greater than 0.5 only for a few cases. Positive correlations of greater importance concern the following correlations Ca/Mg; Cu/K, Mg, Zn; Mg/Fe; K/Mg, Mn, Zn; Mg/Mn. It can be seen that there are almost no negative correlations. In the control group, significant positive correlations concern the following pairs of elements: Cr/Mn; Cu/Mg; K/Mg; Na/Ca. The others are on a level less than 0.5.

### 3.3. Statistically Significant Differences between the Alcohol-Use Disoder (AUD) and Control Groupd

**Figure 4:**
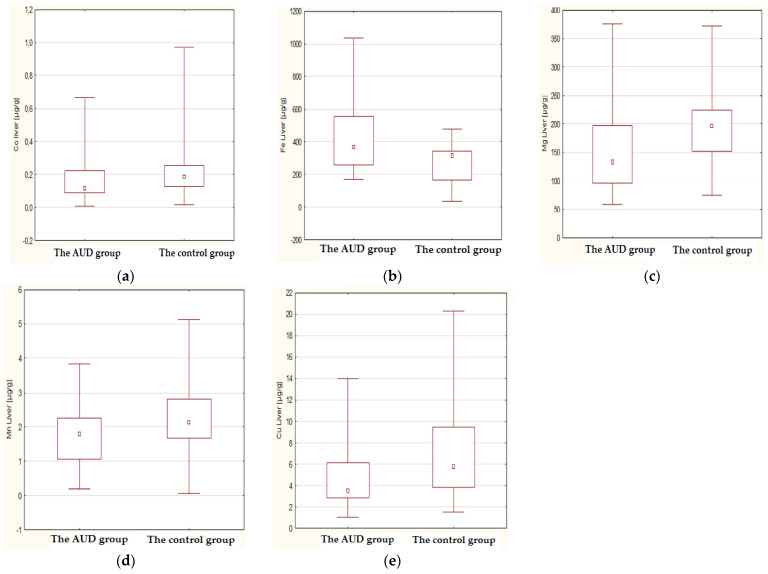
Box and whisker plots considering the median values constructed for statistically significant differences in elements content in the liver tissue of cases (AUD) in comparison to the controls: Co (**a**), Fe (**b**), Mg (**c**), Mn (**d**), Cu (**e**). The Mann–Whitney U test parameters are collected in Table 4.

The Mann–Whitney U test revealed the existence of significant differences in the concentration of several elements, such as Co, Cu, Fe, Mg, and Mn, between the group of chronic alcohol users and the control group (Table 4). The concentration of Co, Cu, Mg, and Mn in the liver of the control group was statistically significantly higher compared to the chronic alcohol consumption group. Only the Fe level was significantly higher in the group of cases as compared to the control. The differences for the remaining elements turned out to be statistically insignificant. The statistically significant differences in the concentration values of the above elements described for both groups are presented in the box-whisker diagrams (Figure 4).

## 4. Discussion

The conducted research shows that the liver tissues in individuals who chronically consume excessive amounts of alcohol contain a significantly lower level of magnesium in comparison to controls. This observation is also confirmed by the works of other authors. The main cause of the decrease in the level of magnesium in the liver tissues is its excessive removal from the body [21]. In 2017, a team of researchers led by Yan Wang [22] demonstrated, in an animal model, the usefulness of magnesium isoglycirizinate (MgIG) in preventing the progression of alcoholic liver disease (ALD). In addition to hepatoprotective activity, MgIG also showed anti-inflammatory and antioxidant properties. The authors argue that MgIG may protect against alcohol-induced liver damage resulting in decreased levels of alanine transaminase and aspartate aminotransferase, and decreased neutrophil infiltration. The authors suggest that the hepatoprotective effect of MgIG is a result of the regulation of neutrophil activity and the alleviation of oxidative stress in the liver. In light of the elemental tests performed, it can be noticed that AUD individuals suffer from magnesium deficiency. Thus, the following question arises: does MgIG act also through the metallic component of the preparation and supplementation of magnesium deficiencies protect the liver from alcohol damage? However, in order to confirm the effect of magnesium by itself, another compound should be tested in this regard, e.g., potassium glycyrrhizinate. Undoubtedly, homeostasis and transport of Mg^2+^ are significantly impaired in liver cells after prolonged exposure to alcohol. The inability of liver cells to accumulate Mg^2+^ after long-term alcohol consumption has been studied mainly using animal models [23,24,25]. It has been reported for years that micronutrient disorders are a common trait in ALD patients. The imbalance of zinc, iron, copper, magnesium and selenium was oftentimes emphasized [26,27]. However, it is the role of magnesium that is most often emphasized as it is proved that magnesium deficiency is associated with liver disease. The deficiency is most likely the result of malabsorption of nutrients, increased urine secretion, low serum albumin or hormone inactivation [28]. Magnesium deficiencies in both serum and liver tissue are dangerous. With low magnesium, mitochondrial function may be disturbed, and there may be defective translocation of protein kinase C (PKC), inflammatory reactions, etc.

It should not be forgotten that the metabolism of ethanol to acetaldehyde in the liver is possible thanks to many enzymes, such as alcohol dehydrogenase (ADH) located in the cytosol, cytochrome P450 2E1 (CYP2E1) in the smooth endoplasmic reticulum, catalase present in peroxisomes. Hepatocytes are able to minimize the toxicity of acetaldehyde by oxidizing it to acetate with the enzyme aldehyde dehydrogenase 2 (ALDH2) inside the mitochondria [29]. However, with chronic ethanol consumption, the metabolism becomes accelerated, hence the elevated levels of ethanol oxidizing enzymes. Thus, in addition to the accumulation of the main product acetaldehyde, enzymes induce the production of reactive oxygen species (ROS) such as free radical forms of ethanol, O_2_^−^, •OH. Due to the overproduction of ROS in relation to the ability to neutralize them by antioxidant enzymes, endogenous and exogenous antioxidants, oxidative stress occurs in the liver cells. As is well known, the antioxidant enzymes that are involved in the neutralization of ROS have micronutrients as co-factors. In view of the observed decrease in the level of Cu or Mn, the decline in the activity of such enzymes as copper-zinc-superoxide dismutase (Cu/Zn-SOD) and manganese-superoxide dismutase (Mn-SOD) as an effect of chronic ethanol administration reported by other authors [30,31,32] seems understandable. The negative effects of alcohol on the processes related to the uptake and absorption of copper have already been described in the literature [33]. For Mn, a decrease in its concentration was observed in the liver of patients in the AUD group compared to the control group. The research of Sassine et al. [34] indicates an increased level of this element in the blood of individuals abusing alcohol. It is understandable due to the fact that Mn was detected in alcoholic beverages, e.g., whiskey and moonshine, where its concentration was determined at the level of 20 µg L^−1^ and 50 µg L^−1^ respectively [35]. This could indicate that the liver, for some reason, does not accumulate this element, despite the increased supply. In the case of Co, there is a certain analogy with Mn. The study showed a lower level of this element in the AUD group compared to the control. It turns out, however, that the probability of an increased supply of this element is high, as even beer may contain from 1 to 1.5 mg of cobalt chloride per liter [36].

It is observed in individuals with chronic alcohol consumption that there are disruptions to iron metabolism in the body. This manifests itself in the form of increased Fe uptake by hepatocytes and Kupffer cells [37,38]. In our work, we observed an increased level of this element, which is consistent with the observations of other authors. Fe accumulation is not a beneficial phenomenon. Fe is involved in the Fenton reaction, inducing the mechanisms of oxidative stress, which results in cell damage. A key role in the processes of Fe absorption is played by decreased expression of heptcidin 1 in the liver [39], increased expression of ferroportin and DMT1 [40], increased expression of the transferrin receptor in ALD patients [41].

Regarding the observed intercorrelations, new strong positive relationships appeared in the AUD group, which were not observed in the control group. Mg strongly positively correlated with Ca, Mn, Fe; K correlated with Mn and Zn, and Cu positively correlated with K and Zn. Although these correlations have not reached statistical significance, they shed new light on the complex processes of homeostasis in trace element metabolism. So far, such intercorrelations have been tracked by our team in body fluids such as fluid from the anterior chamber of the human eye [42,43], and in tissues collected post mortem from various sites of the human brain [12,13,44,45]. Our research to date has confirmed that in the case of disturbance of physiological processes that take place in eye diseases (cataracts), AUD, or are the result of medical procedures such as hemodialysis [44], there are visible changes in the intercorrelation matrices. In the present study, it can be seen that most elements evaluated in tissues collected from the AUDs of patients are related to each other by strong positive correlations represented by Spearman’s r coefficient. Most of the above correlations are not statistically significant. Besides, they do not reflect the intercorrelations described in the literature in the process of absorption of elements. For example, the absorption of Mg in the intestine via para-cellular transport is in opposition to Ca ions [45]. In our study, a fairly strong positive correlation between these elements was found in the liver tissues. It should be emphasized, however, that there are a number of controversies on this issue and there is no clear consensus so far. Rayssiguier et al. [45] reported an increase in the level of Ca in the liver with the occurrence of hypomagnesaemia. In turn, Leevy and Moroianu [46] observed a decrease in Ca levels in alcohol abusers, which is consistent with the study of Pitts and Van Thiel [47].

In our study, the decrease in Mg level strongly correlates with the level of Mn (0.84). This is the strongest positive correlation that occurs in the AUD group. This means that if the Mg level decreases, the Mn level also proportionally decreases. This may be due to elimination or compensation mechanisms for Mn, which seems to be excessively toxic. Mn absorption is also likely to be impaired by the observed elevated Fe levels [48], because in the physiological state both Mn and Fe compete for transferrin binding. Both metals possess a high affinity to transferrin receptors (TfR) [49]. However, this issue requires further clarification, especially as Mn is an important trace element necessary for the work of many enzymes (Mn-superoxide dismutase, glutamine synthetase, arginase, glycosyltransferase and xylosyltransferase, isocitrate dehydrogenase, serine/threonine phosphatase, pyruvate carboxylase, phosphoenolpyruvate carboxykinase), and the liver is the main organ of its accumulation [50].

## 5. Conclusions

Knowledge concerned with the role of micronutrients in the pathogenesis and progression of ALD is still expanding, but there are many aspects that require confirmation and clarification. The problem is complicated by the fact that micronutrients often act as co-enzymes of numerous biochemical enzymes responsible for inflammatory reactions, oxidative stress, and proliferation. Explanation of the mechanisms of pathophysiology related to alcohol abuse should be preceded by elemental mapping. This is a prerequisite to proposing rational intervention in clinical practice. Several reviews of metallomics in the context of alcoholism have appeared in recent years [13,15,17,27,47,48,51,52]. However, there are still many limitations to the research undertaken related to the small sample size, discrepancies in the measurement techniques used, the lack of homogeneity of the samples, etc. Most of the studies, especially those on the elemental composition of tissues, were carried out using animal models. Therefore, the observations made should be verified on human tissues collected from patients from various environments. Our study confirmed the existence of significant differences between the AUD and control groups in the levels of Co, Cu, Fe, Mg, and Mn. Except for the elevated Fe level, the remaining elements were characterized by a reduced level in the liver tissues of individuals exposed to chronic alcohol consumption as compared to the controls. There are also quite strong inter-elemental correlations, which in the control group concern only a few pairs of elements Cr-Mn, Cu-Mg, K-Mg, while in the AUD group these series have been expanded into the following strong positive intercorrelations Mg-Ca, Mn, Fe; K-Mn, Zn; Cu-K, Zn. The strongest positive correlation distinguishing the AUD group is the correlation of the Mg-Mn pair (0.87). However, the clarification of this observation, as well as the potential influence of Fe on this inter-elemental relationship, requires further research on a larger study group and the inclusion of additional biological material, i.e., blood and urine.

## Figures and Tables

**Table 1:** Demographic characteristic of the patients’ groups enrolled in the study.

Group	Controls (n = 45)	Cases (n = 39)	*p*
Mean age ± SD	50.33 ± 19.10	47.89 ± 13.32	0.413
Gender (n/%)	female: 14 (31.11%)	female: 10 (25.64%)	0.579
	male: 31 (68.89%)	male: 29 (74.36%)	
Mean weight [kg]	81.955 ± 20.944	81.358 ± 25.438	0.642
BMI [kg m^−2^] (mean ± SD)	28.456 ± 7.32	26.512 ± 7.41	0.650

Abbreviations: Body mass index—BMI.

**Table 2:** Applied wavelengths (λ), the detection limits (LOD), together with the correlation coefficient of the calibration curve (R) achieved for the examined elements.

Element	Λ [nm]	LOD [mg L^−1^]	R
Mg	279.553	0.01	0.9996
K	766.491	0.2	0.9996
Na	589.592	0.1	0.9997
Ca	422.673	0.01	0.9998
Cr	267.716	0.2	1.0000
Zn	213.857	0.1	0.9999
Co	238.892	0.3	0.9999
Mn	257.610	0.03	1.0000
Cu	327.395	0.15	1.0000
Se	196.026	2.0	0.9999
Fe	238.204	0.2	1.0000

**Table 3:** A descriptive statistic of average liver trace element concentrations in the alcohol use disorder (AUD) group and control group. Abbreviations: standard error (SE), standard deviation (SD), group size (N). The Mann–Whitney test was used to compare central tendency of both groups.

Element	The AUD Group							The Control Group						
	N	Mean [µg/g]	Median [µg/g]	Min [µg/g]	Max [µg/g]	SD	SE	N	Mean [µg/g]	Median [µg/g]	Min [µg/g]	Max [µg/g]	SD	SE
Ca	35	181.415	166.990	59.105	662.016	121.220	20.490	45	199.943	160.422	12.601	513.214	98.153	14.631
Co	31	0.155	0.117	0.011	0.667	0.131	0.024	43	0.221	0.185	0.019	0.972	0.158	0.024
Cr	35	0.142	0.133	0.066	0.308	0.056	0.009	44	0.162	0.146	0.056	0.434	0.072	0.011
Cu	36	4.561	3.532	1.041	13.965	2.852	0.473	45	7.274	5.731	1.541	20.310	4.627	0.690
Fe	39	415.811	365.519	170.468	1037.217	197.492	31.624	31	267.058	315.300	36.368	480.465	125.073	22.464
K	36	1413.296	1361.149	749.301	3359.705	537.728	89.621	45	1507.900	1567.416	149.818	2506.878	470.254	70.101
Mg	36	149.136	133.003	59.156	375.039	73.063	12.177	45	196.713	196.362	75.629	372.551	64.797	9.659
Mn	36	1.797	1.791	0.193	3.846	0.859	0.143	45	2.318	2.118	0.066	5.122	1.175	0.171
Na	36	827.503	840.872	288.063	1630.992	321.576	53.596	45	916.410	805.901	31.939	2515.700	431.902	64.384
Se	29	0.651	0.610	0.117	1.636	0.385	0.071	36	0.469	0.460	0.017	1.181	0.305	0.051
Zn	36	78.048	56.925	8.822	288.344	63.526	10.5878	44	87.267	88.918	4.089	198.083	41.251	6.219

**Table 4:** Differences between controls and cases considering selected elemental ratios. Analysis was performed by the Mann–Whitney U test.

	Sum.Rank AUD	Sum.Rank Control	U	Z	*p*	N AUD	N Control
Ca	1279.0	1961.0	649.0	−1.34325	0.179191	35	45
Co	901.5	1873.5	405.5	−2.85947	0.004244	31	43
Cr	1263.0	1897.0	633.0	−1.35209	0.176347	35	44
Cu	1160.0	2161.0	494.0	−3.00340	0.002670	36	45
Fe	1631.0	854.0	358.0	2.91451	0.003563	39	31
K	1345.0	1976.0	679.0	−1.24508	0.213103	36	45
Mg	1134.0	2187.0	468.0	−3.25052	0.001152	36	45
Mn	1252.0	2069.0	586.0	−2.12899	0.033256	36	45
Na	1393.0	1928.0	727.0	−0.78887	0.430190	36	45
Se	1098.0	1047.0	381.0	1.86075	0.062781	29	36
Zn	1260.0	1980.0	594.0	−1.91485	0.055512	36	44

Abbreviations: Sum.rank—the sum of ranks, U—original Mann–Whitney statistic, distribution of which is well described for small N, Z—standardized U statistic with standard normal distribution (well described for all N), Z-corrected—Z statistic with correction for continuity, N—number of observations.

## Data Availability

The data presented in this study are available upon request from Jacek Baj.
